# Epicatechin Stimulates Mitochondrial Activity and Selectively Sensitizes Cancer Cells to Radiation

**DOI:** 10.1371/journal.pone.0088322

**Published:** 2014-02-06

**Authors:** Hosam A. Elbaz, Icksoo Lee, Deborah A. Antwih, Jenney Liu, Maik Hüttemann, Steven P. Zielske

**Affiliations:** 1 Department of Radiation Oncology, Wayne State University, Detroit, Michigan, United States of America; 2 Center for Molecular Medicine and Genetics, Wayne State University, Detroit, Michigan, United States of America; 3 Cardiovascular Research Institute, Wayne State University, Detroit, Michigan, United States of America; 4 Wayne State University and Karmanos Cancer Institute, Detroit, Michigan, United States of America; 5 College of Medicine, Dankook University, Cheonan-si, Chungcheongnam-do, Republic of Korea; Technische Universitaet Muenchen, Germany

## Abstract

Radiotherapy is the treatment of choice for solid tumors including pancreatic cancer, but the effectiveness of treatment is limited by radiation resistance. Resistance to chemotherapy or radiotherapy is associated with reduced mitochondrial respiration and drugs that stimulate mitochondrial respiration may decrease radiation resistance. The objectives of this study were to evaluate the potential of (-)-epicatechin to stimulate mitochondrial respiration in cancer cells and to selectively sensitize cancer cells to radiation. We investigated the natural compound (-)-epicatechin for effects on mitochondrial respiration and radiation resistance of pancreatic and glioblastoma cancer cells using a Clark type oxygen electrode, clonogenic survival assays, and Western blot analyses. (-)-Epicatechin stimulated mitochondrial respiration and oxygen consumption in Panc-1 cells. Human normal fibroblasts were not affected. (-)-Epicatechin sensitized Panc-1, U87, and MIA PaCa-2 cells with an average radiation enhancement factor (REF) of 1.7, 1.5, and 1.2, respectively. (-)-Epicatechin did not sensitize normal fibroblast cells to ionizing radiation with a REF of 0.9, suggesting cancer cell selectivity. (-)-Epicatechin enhanced Chk2 phosphorylation and p21 induction when combined with radiation in cancer, but not normal, cells. Taken together, (-)-epicatechin radiosensitized cancer cells, but not normal cells, and may be a promising candidate for pancreatic cancer treatment when combined with radiation.

## Introduction

Radiotherapy is ideal for many solid tumors because of its localized cytotoxic effect. Radiation resistance, however, is a common problem which is responsible for recurrence of tumors in cancer patients [1]. The Warburg effect, in which mitochondrial respiration is suppressed even in the presence of oxygen, and aerobic glycolysis is stimulated, is believed to mediate resistance to chemotherapy and radiotherapy in solid tumors [2], [3]. Treatment with ionizing radiation stimulates mitochondrial respiration and increases reactive oxygen species (ROS) production in cancer cells [4].

Previous reports have suggested a reduced risk of cancer in patients regularly consuming fruits and vegetables [5]. Flavonoids are a ubiquitous class of polyphenolic compounds that are present in fruits and vegetables and comprise several groups of compounds such as flavanols, flavones, flavonols, isoflavones, flavanones, anthocyanidins, and proanthocyanidins [4], [6]. A number of beneficial health effects, such as cancer prevention are linked to flavonoids in the diet [7]. Moreover, flavonoids were shown to sensitize cancer cells to chemotherapy and radiotherapy, but more often have been shown to exhibit radioprotective effects on normal tissues [8]–[11]. (-)-Epicatechin is a monomeric flavanol that is a natural compound found in many fruits and vegetables, in particular in cocoa and green tea [6], [12], and it exhibits several beneficial effects to human health [13]. A recent study by our group showed that (-)-epicatechin stimulated mitochondrial respiration and increased mitochondrial mass in mice [14].

The ability of the flavanol (-)-epicatechin to stimulate mitochondrial respiration and increase mitochondrial mass in a mouse model [14], together with an earlier study showing that radiation stimulates mitochondrial respiration in cancer cells [4], lead us to hypothesize that (-)-epicatechin may sensitize cancer cells to radiotherapy, because both counteract Warburg metabolism. The objectives of this study were to examine the ability of (-)-epicatechin to stimulate mitochondrial respiration in cancer cells and to examine the ability of (-)-epicatechin to selectively sensitize cancer cells to radiation. We here show that (-)-epicatechin stimulated cytochrome *c* oxidase (COX) activity and mitochondrial respiration in pancreatic cancer cells. In addition, (-)-epicatechin sensitized pancreatic cancer and glioblastoma cells, but not normal fibroblasts, to radiation. (-)-Epicatechin in combination with ionizing radiation stimulated Chk2 (checkpoint kinase 2) phosphorylation, p21 expression, and increased apoptosis, in cancer cells. These results suggest that (-)-epicatechin exhibits the potential to improve the therapeutic outcome for cancer patients by augmenting conventional radiotherapy.

## Materials and Methods

### (-)-Epicatechin

(-)-Epicatechin was obtained from Sigma-Aldrich as compound HPLC-purified from green tea (≥98% purity) (#E4018). A 2 mM stock solution of (-)-epicatechin was made in PBS and aliquots were stored at −80°C. The results of freshly prepared (-)-epicatechin were consistent with −80°C stocks over the course of this study. Testing for generation of reactive oxygen species in cells by (-)-epicatechin-breakdown products after storage at −80°C was negative. Sodium pyruvate in DMEM used for cell culture serves as an effective scavenger of potential oxidants from nutraceuticals such as (-)-epicatechin [15].

### Cells and cell culture

Panc-1 cells, MIA PaCa-2 cells, U87 cells, and human normal fibroblasts were purchased from the American Type Culture Collection and used at low passage (Manassas, VA). U87 cells are a model cell line for human glioblastoma-astrocytoma. Panc-1 cells and MIA PaCa-2 cells are model cell lines for human pancreatic epithelial carcinoma. Fibroblasts are a model cell line for normal human cells. Cells were cultured and maintained in Dulbecco's Modified Eagle's Medium (DMEM) (high glucose, with pyruvate) supplemented with 10% fetal bovine serum (FBS) and 2 mM L-glutamine in absence of antibiotics. Fibroblasts were passaged after dissociation with 0.05% trypsin/EDTA and other cell lines were dissociated with 0.25% trypsin/EDTA. Cell lines were routinely screened for mycoplasma contamination and found to be negative.

### Oxygen consumption

Oxygen consumption was assayed by measuring COX activity which accounts for 90% of all cellular oxygen consumption. COX activity was analyzed in Panc-1 cells with a micro-Clark-type oxygen electrode in a closed chamber (Oxygraph System; Hansatech, Norfolk, UK) at 25°C. Panc-1 cells were seeded into T-150 flasks and on the next day, cells were treated with different concentrations of (-)-epicatechin for 1 h, then harvested and solubilized in 10 mM HEPES (pH 7.4), 40 mM KCl, 1%Tween-20, 1 µM oligomycin, 1 mM PMSF,10 mM KF, 2 mM EGTA, and 1 mM Na_3_VO_4_. COX activity was measured in the presence of 20 mM ascorbate and 200 µM substrate cytochrome *c* from cow heart (Sigma-Aldrich). Oxygen consumption was recorded on a computer and analyzed with the Oxygraph software. Protein concentration was determined with the DC protein assay kit (Bio-Rad, Hercules, CA,). COX activity is defined as nanomols O_2_ consumed per minute per milligram total protein.

### Clonogenic survival assay

Cells were seeded into T-25 flasks at 5×10^5^ cells per flask. On the next day, cells were exposed to 0–200 µM (-)-epicatechin for 1 h and then irradiated with 0–8 Gy with a Pantak 320 kV orthovoltage unit at 0.86 Gy per min and 10 mA. Twenty-four hours after irradiation, cells were trypsinized, counted, and plated at predetermined clonal densities. Two weeks later, cells were fixed with a methanol/acetic acid mixture (7∶1) and stained with crystal violet. Colony counting was done manually and data were analyzed by determining the surviving fraction at each dose of radiation. Cell survival curves were fit to a linear-quadratic model and radiation enhancement factors (REF) are derived as the area under the control (no drug) curve divided by the area under the test (with drug) curve [16].

### Western blot analysis

Cells were exposed to 0–200 µM (-)-epicatechin for 1 h and then irradiated with 0 or 6 Gy. Twenty-four hours after irradiation, cells were harvested and lysed in 1% SDS, Halt Protease and Phosphatase Inhibitor Cocktail, Phosphatase Inhibitor Cocktail 3 (Sigma, Cat. No. P 0044), Phosphatase Inhibitor Cocktail 2 (Sigma, Cat. No. P 5726), 10 mM, pH 7.4, 2 mM Na_3_VO_4_, 1 mM NaF, and 1.7 mM Na_4_P_2_O_7_. Protein content was determined using a BCA assay (Thermo Scientific). Samples for phospho-Chk2 (Thr68) and p21 were denatured by boiling with 2× Laemmli buffer supplemented with 10% β-mercaptoethanol, while samples for mitochondrial proteins were mixed with 2× Laemmli buffer supplemented with 10% β-mercaptoethanol and shaken on a rocker at room temperature for 30 min. Thirty µg of protein was loaded per well using 4–20% mini-PROTEAN® TGX™ precast gels (Bio-Rad) and then transferred to PVDF membranes using a Trans-Blot Turbo Plus system (Bio-Rad). After transfer, membranes were blocked with 5% non-fat dry milk for 1 h at room temperature then incubated overnight with primary anti-P-Chk2 (Thr68), anti-p21, anti-caspase 3 anti-β-actin, anti-GAPDH (1∶1000–1∶5000; Cell Signaling Technologies, Boston, MA), oxidative phosphorylation (OxPhos) complex I NDUFB6 subunit, OxPhos complex II subunit 70 kDa Fp, OxPhos complex III core subunit I, cytochrome c, OxPhox complex IV subunit I, or OxPhos complex V α subunit (1∶2000–1∶8000; MitoSciences, Eugene, OR). Membranes were washed with Tris buffered saline with 0.1% Tween-20 (TBS-T), incubated with secondary anti-mouse or anti-rabbit antibodies (1∶4000; Cell Signaling Technologies) washed again and incubated with SuperSignal West Pico Chemiluminescent Substrate for exposure to x-ray film. Protein bands were quantitated using Quantity One version 4.6.7 (Bio-Rad).

### Statistical analysis

All experiments were repeated at least three times. Clonogenic experiments were conducted in triplicate. Results are presented as mean ± SEM. Statistical analyses were performed using GraphPad Prism version 6 (GraphPad Software for Science, Inc, San Diego, CA) with the Mann-Whitney test, Student's t-test, or one-way ANOVA, where appropriate. Post-hoc Bonferroni's multiple comparisons test was conducted on significant ANOVA results. Results are considered significant when p<0.05. Error bars represent ± SEM.

## Results

### (-)-Epicatechin stimulates mitochondrial respiration

**Figure 1:**
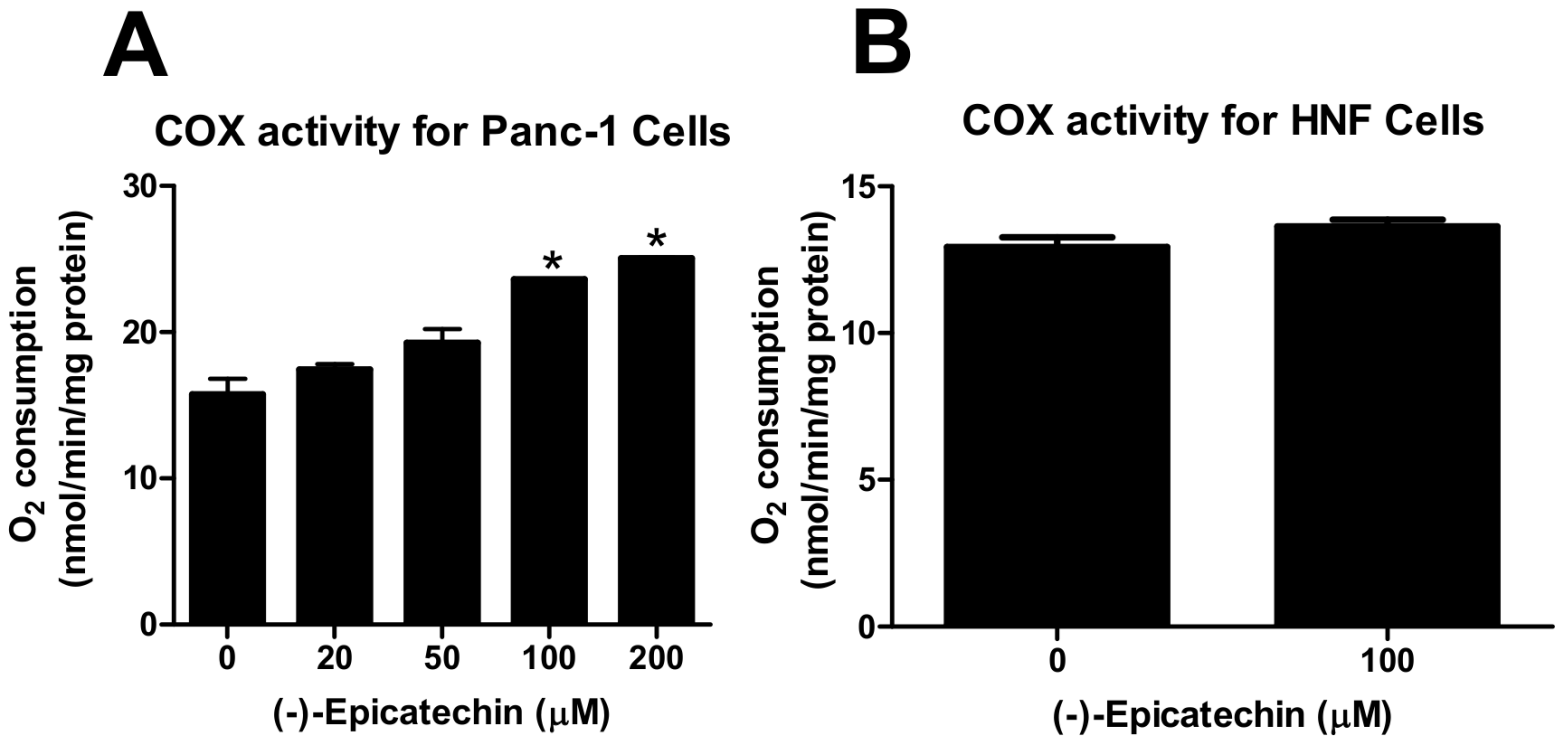
(-)-Epicatechin is a flavanol that selectively stimulates mitochondrial respiration in pancreatic cancer cells. (A) Oxygen consumption by cytochrome c oxidase (COX) from Panc-1 cells exposed to different (-)-epicatechin concentrations. Turnover is defined as consumed O_2_ (nM)/(min · total protein (mg)). Respiration rates are increased by (-)-epicatechin in a concentration-dependent manner. *p<0.05, compared to 0 µM (-)-epicatechin. (B) Oxygen consumption by cytochrome c oxidase (COX) from human normal fibroblast (HNF) cells exposed to 0 or 100 µM (-)-epicatechin. Respiration rates are not increased by 100 µM (-)-epicatechin.

We have previously shown that (-)-epicatechin stimulates mitochondrial respiration in normal mouse muscle tissues [14]. To examine whether (-)-epicatechin could stimulate mitochondrial respiration in cancer cells, we examined the activity of COX by evaluating the rate of oxygen consumption in Panc-1 cells. COX transfers electrons from cytochrome *c* (Cytc) to molecular oxygen, and ‘charges’ the mitochondria by pumping protons across the inner mitochondrial membrane that are used later for ATP generation. The Cytc/COX reaction is the proposed rate-limiting step of the electron transport chain in vivo [17], [18], thus controlling the overall rate of cellular respiration. (-)-Epicatechin stimulated COX activity in a dose dependent manner in pancreatic cancer cells when used at concentrations of up to 200 µM (Figure 1A, p<0.05). At 200 µM (-)-epicatechin, mitochondrial respiration was increased by 59% from 15.7 to 25.0 nmol O_2_/min per milligram protein.

To determine whether stimulation of mitochondrial respiration by (-)-epicatechin could occur in normal, non-cancerous cell types, we treated human normal fibroblasts (HNF) with epicatechin and measured COX activity. No significant increase in O_2_ consumption was observed after 100 µM treatment (Fig. 1B). These data show that (-)-epicatechin selectivity stimulates mitochondrial respiration in cancer cells.

### (-)-Epicatechin sensitizes cancer cells to ionizing radiation

**Figure 2:**
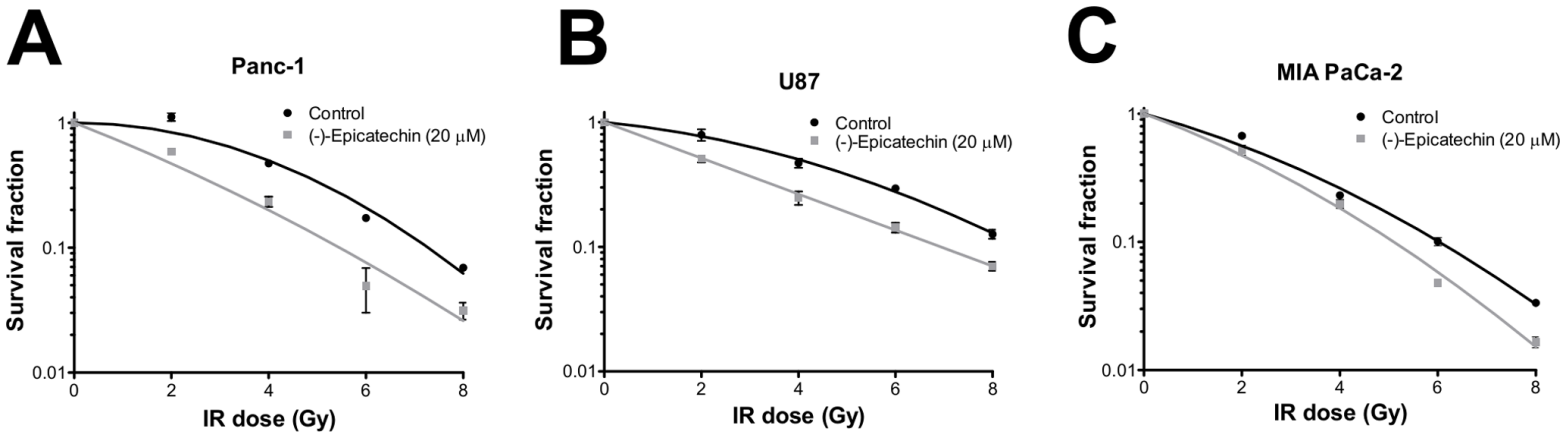
(-)-Epicatechin sensitizes cancer cells to radiation. (A) (-)-Epicatechin sensitizes Panc-1 cells to radiation with an average REF = 1.7 (B) (-)-Epicatechin sensitizes U87 cells to radiation with an average REF of 1.5. (C) (-)-Epicatechin sensitizes MIA PaCa-2 cells to radiation with an average REF = 1.2.

Increased mitochondrial respiration due to (-)-epicatechin treatment may reverse Warburg metabolism and thus sensitize cancer cells to radiation. To test this, we examined the effect of (-)-epicatechin on clonogenic survival of Panc-1, U87, and MIA PaCa-2 cancer cell lines. (-)-Epicatechin significantly enhanced radiosensitivity in Panc-1 cells at doses from 2 to 8 Gy (p = 0.0001, Fig. 2A). In U87 cells, (-)-epicatechin also significantly enhanced radiosensitivity at doses from 2 to 8 Gy (p = 0.0001, Fig. 2B). Similar results were found in the MIA PaCa-2 cell line, in which (-)-epicatechin produced significant radiosensitization at each dose tested (p = 0.0001, Fig. 2C). Cell survival curves were fit to a linear quadratic model. In Panc-1 cells, the surviving fraction at 2 Gy was 98%, whereas (-)-epicatechin treatment significantly reduced the the surviving fraction to 58% (p = 0.001). In U87 cells, the surviving fraction at 2 Gy was 79% and (-)-epicatechin significantly reduced survival to 51% (p = 0.021). In MIA PaCa-2 cells, the surviving fraction at 2 Gy was 67%, whereas (-)-epicatechin treatment significantly reduced the surviving fraction at 2 Gy to 52% (p = 0.029). The enhancement of radiation-induced cell death or radiation enhancement factor (REF) was 1.7 for Panc-1, 1.5 for U87, and 1.2 for MIA PaCa-2 cells. These data show that (-)-epicatechin radiosensitizes cancers of multiple types in vitro.

### (-)-Epicatechin does not sensitize HNF cells to radiation

**Figure 3:**
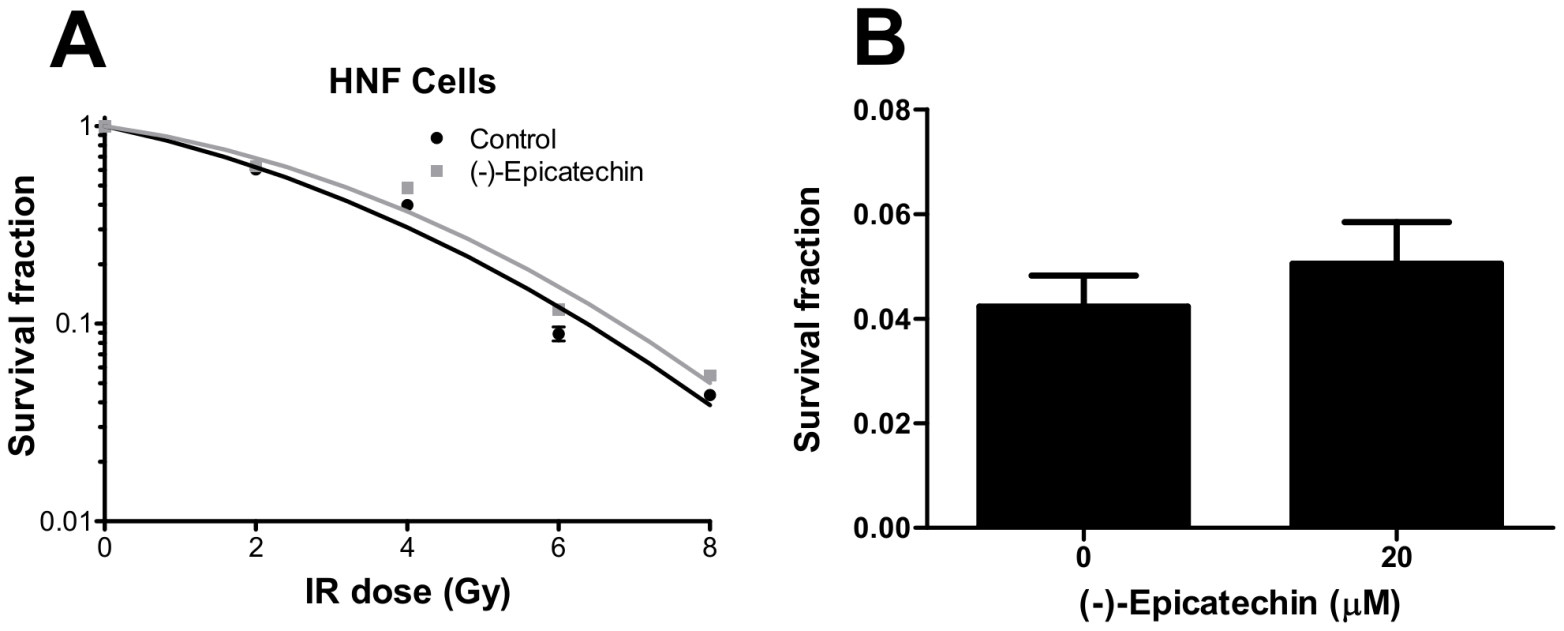
(-)-Epicatechin does not sensitize normal cells to radiation. (A) 20 µM (-)-Epicatechin does not sensitize HNF cells to radiation (REF = 0.9). (B) (-)-Epicatechin alone does not inhibit colony formation in HNF (p>0.05).

To test whether (-)-epicatechin radiosensitization is selective for cancer cells, we examined the effect of (-)-epicatechin on HNF cells. (-)-Epicatechin at 20 µM did not sensitize HNF cells to radiation with a REF of 0.9 (Figure 3A). Neither did (-)-epicatechin alone decrease clonogenic survival of HNF cells (Figure 3B). These data show that (-)-epicatechin is selective for cancer cells and thus may not increase normal tissue toxicity when combined with radiation.

### Effect of (-)-epicatechin on electron transport chain (ETC) protein expression

**Figure 4:**
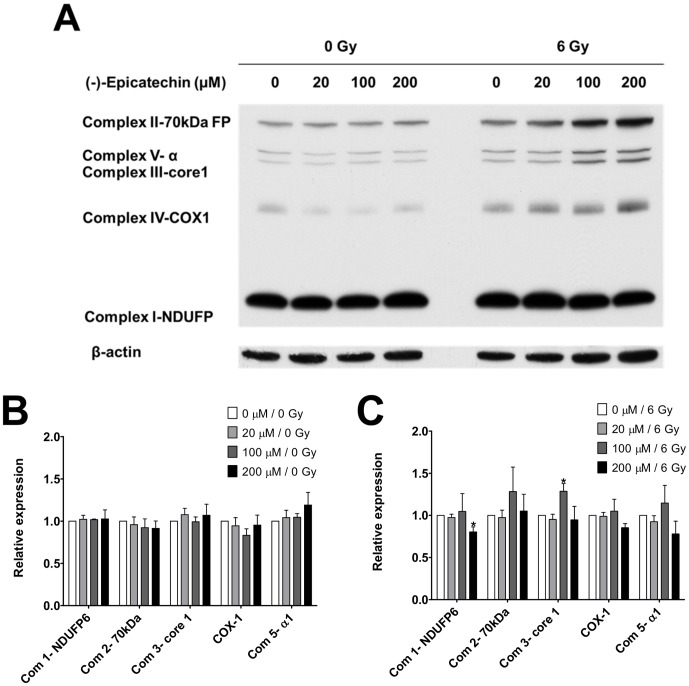
The effect of (-)-epicatechin on ETC protein expression in combination with radiation. (A) A representative blot for ETC protein expression in Panc-1 cells that were treated with 0–200 µM (-)-epicatechin and 0 or 6 Gy. (B) (-)-Epicatechin alone does not significantly change ETC protein expression. Protein levels are normalized to the untreated control. (B) (-)-Epicatechin induced minimal changes in ETC protein expression when combined with 6 Gy. Values were normalized to 6 Gy alone, and * p<0.05 relative to 6 Gy/0 µM (-)-epicatechin.

Previous studies showed that increased mitochondrial respiration via (-)-epicatechin application is associated with a small but significant upregulation of ETC protein complexes in vivo [14]. We performed Western blot analysis for all OxPhos complexes using antibodies for a key subunit of each complex (NDUFP, 70 kDa FP, core1, COX1, ATP synthase α) in Panc-1 cells treated with 0–200 µM (-)-epicatechin prior to exposure to 0 or 6 Gy. (-)-Epicatechin alone did not significantly change the expression of any of the ETC proteins in Panc-1 cells (Figure 4A–B). Combining (-)-epicatechin with 6 Gy had a modest effect on complex I (NDUFP) at 200 µM and complex III core subunit 1 at 100 µM (Figure 4A and C, p = 0.0006). These data suggest that (-)-epicatechin, in this cancer cell line, does not trigger large changes in mitochondrial OxPhos complexes levels or mediate radiosensitization through large changes in the levels of ETC regulatory subunits.

### (-)-Epicatechin and radiation stimulate checkpoint kinase 2 phosphorylation and p21 expression in Panc-1 but not HNF cells

**Figure 5:**
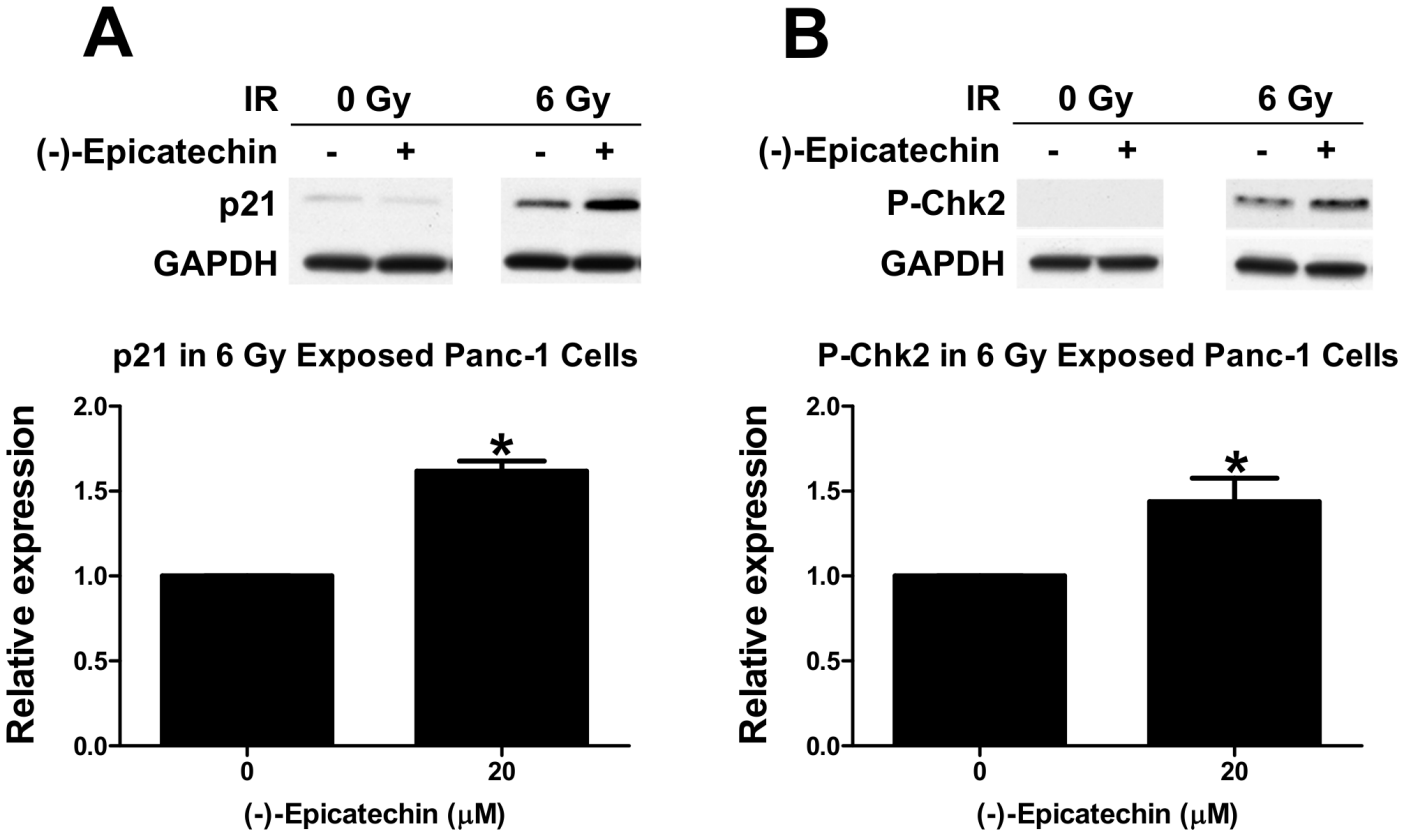
(-)-Epicatechin stimulates checkpoint kinase protein 2 phosphorylation at threonine 68 and p21 expression in Panc-1 cells. (A) (-)-Epicatechin stimulates p21 expression. Protein levels in 20 µM (-)-epicatechin treated samples were normalized to samples treated with 6 Gy. (B) 20 µM (-)-epicatechin stimulates Chk2 phosphorylation. Protein levels in (-)-epicatechin treated samples were normalized to samples that were treated with 6 Gy, and *p<0.05.

Phosphorylation of checkpoint kinase protein 2 (p-Chk2) at threonine 68 and the overexpression of p21 are implicated in DNA damage response and radiosensitization [19], [20]. We analyzed p21 expression and Chk2 phosphorylation in Panc-1 cells treated with 0 or 20 µM (-)-epicatechin after exposure to 0 or 6 Gy. (-)-Epicatechin alone did not cause any change in p21 levels or Chk2 phosphorylation 24 h after treatment. However, combination of (-)-epicatechin and 6 Gy treatment stimulated p21 expression (Figure 5A, p = 0.0005) and Chk2 phosphorylation (Figure 5B, p = 0.042). The expression of p21 was increased 62% and P-Chk2 (Thr68) levels increased 43% in irradiated cells treated with (-)-epicatechin, compared to cells receiving radiation alone.

**Figure 6:**
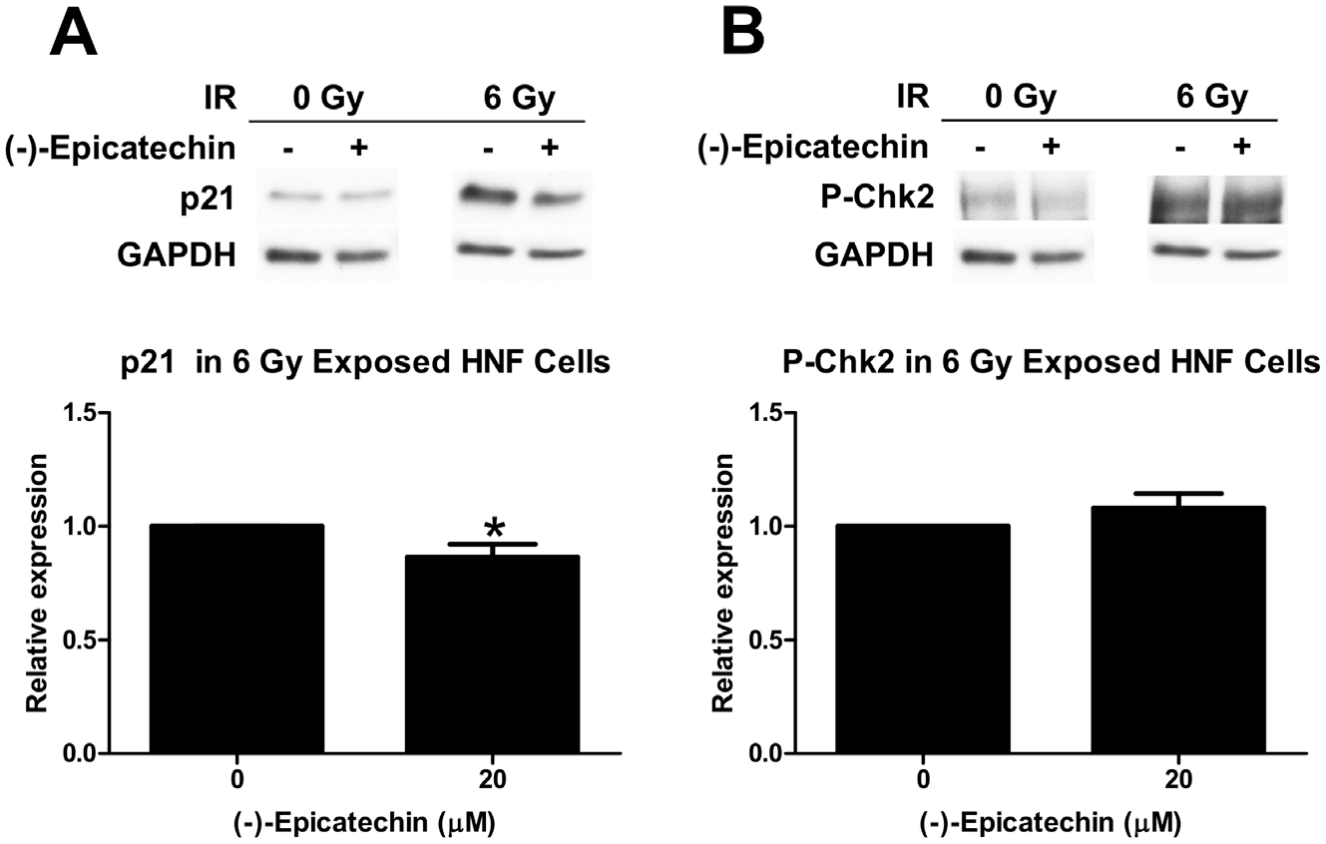
(-)-Epicatechin inhibits p21 expression and does not stimulate checkpoint kinase protein 2 phosphorylation at threonine 68 in HNF cells. (A) (-)-Epicatechin inhibits p21 expression. Protein levels in 20 µM (-)-epicatechin treated samples were normalized to samples treated with 6 Gy, and *p<0.05. (B) 20 µM (-)-epicatechin does not stimulate Chk2 phosphorylation. Protein levels in (-)-epicatechin treated samples were normalized to samples that were treated with 6 Gy.

We then investigated P-Chk2 and p21 in HNF cells after treatment with (-)-epicatechin and radiation since HNF cells are not radiosensitized (Fig. 3). In contrast to Panc-1 cancer cells, combination (-)-epicatechin plus 6 Gy treatment reduced p21 expression by 15% (Fig. 6A, p = 0.03). Chk2 phosphorylation was unchanged (Fig. 6B). These data suggest that (-)-epicatechin radiosensitizes cancer cells by perturbations of cell cycle and checkpoint responses to radiation.

### (-)-Epicatechin and radiation stimulate cleavage of caspase 3 in Panc-1 cells

**Figure 7:**
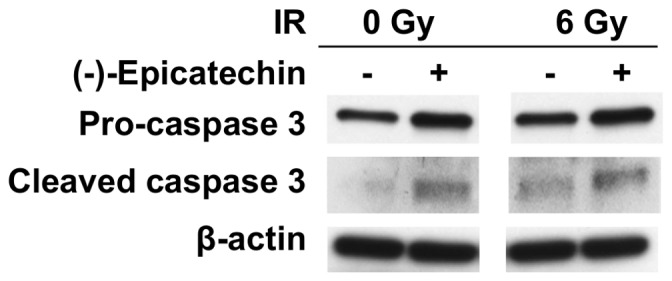
(-)-Epicatechin stimulates caspase 3 cleavage and expression in Panc-1 cells. Panc-1 cells were exposed to 0 or 20 µM (-)-epicatechin for 1 hour, then exposed to 6 Gy of radiation, and incubated for 72 hours. (-)-Epicatechin stimulates pro-caspase 3 expression and cleavage.

Caspase 3 is critical for execution of apoptosis. To examine the possibility that (-)-epicatechin sensitizes cancer cells to radiation by inducing apoptosis, we performed Western blot analysis for pro-caspase 3 and cleaved caspase 3 in Panc-1 cells exposed for 72 hours to 0 or 20 µ (-)-epicatechin and 0 or 6 Gy of radiation. Panc-1 cells exposed to (-)-epicatechin showed increased pro-caspase 3 expression and cells treated (-)-epicatechin and/or 6 Gy showed increased caspase 3 cleavage compared to samples exposed to 6 Gy alone (Fig. 7). These data suggest that (-)-epicatechin radiosensitizes pancreatic cancer cells by stimulating caspase 3 expression and apoptosis.

## Discussion

(-)-Epicatechin stimulated mitochondrial respiration in Panc-1 cells as evident by increased oxygen consumption rates. While (-)-epicatechin sensitized glioblastoma and pancreatic cancer cells to radiation, it did not sensitize HNF cells, indicating that the radiosensitization effect of (-)-epicatechin is specific to cancer cells. (-)-Epicatechin stimulated mitochondrial respiration and exhibited radiosensitization in Panc-1 cells in a dose dependent manner. Combining (-)-epicatechin with 6 Gy stimulated Chk2 phosphorylation, p21 expression, and induced greater cleavage of caspase 3. These effects were observed preferentially in cancer cells but not normal cells (HNF).

The results shown herein are novel and significant in three respects. Firstly, (-)-epicatechin sensitized pancreatic cancer cells to radiation. Pancreatic cancer is typically resistant to conventional therapeutic interventions [21] and radiosensitizing drugs may thus be useful to overcome this therapeutic hurdle. Secondly, most types of pancreatic cancer are characterized by Kras mutations [22] and our results showed that (-)-epicatechin radiosensitizes Kras mutant Panc-1 and MIA PaCa-2 cell lines. Thirdly, we showed that (-)-epicatechin sensitized cancer cells but not HNF cells to radiation, which is consistent with earlier studies and indicates cancer cell selectivity [9], [10], [23]. Moreover, the novelty of our results is evident in showing stimulated mitochondrial respiration in cancer cells at concentrations that are more therapeutically relevant and that have not been tested in pancreatic cancer cells. Bioavailability of (-)-epicatechin when administered orally as part of a food extract such as green tea or cocoa may be an issue because of limited gut absorption. In this study, we used purified (-)-epicatechin compound and envision a more direct route of therapeutic administration in vivo that may allow for greater plasma concentrations. Notably, our previous studies have found biological effects after oral administration of pure compound [14]. Although this study showed an effect of (-)-epicatechin on normal tissue, while the current study does not, differences in the models and cell types studied are profound and may account for this discrepancy. Metabolism, signaling, and ETC isoforms are likely different between muscle cells and fibroblasts.

Stimulating mitochondrial respiration in pancreatic cancer cells may not necessarily require increased ETC protein expression. Numerous studies have shown that COX is decisively regulated by phosphorylation on both the catalytic and regulatory subunits [24]. Therefore, stimulation of mitochondrial respiration by (-)-epicatechin in cancer cells could be mediated by a signaling mechanism that remains to be elucidated. (-)-Epicatechin was shown to inhibit MAPK/Erk [25], [26] and MAPK was shown to associate with EGFR and translocate to the mitochondria where it binds to COX [27] suggesting a potential signaling mechanism for the (-)-epicatechin-mediated regulation of mitochondrial respiration.

Combining (-)-epicatechin with radiation induced Chk2 phosphorylation in Panc-1 cells. Chk2 phosphorylation at threonine 68 occurs following exposure to radiation as part of the DNA damage response [28], [29]. Several studies show the involvement of p21 in radiosensitization in several types of cancer [19], [20], [30]. Moreover, studies show that the phosphorylation of Chk2 at threonine 68 and increased p21 expression reduces cell proliferation by inducing senescence [31]–[33]. The ability of (-)-epicatechin to stimulate Chk2 phosphorylation and p21 expression could potentially explain, at least in part, a mechanism by which (-)-epicatechin causes radiosensitization and inhibition of clonogenic survival in Panc-1 cells. In additional, (-)-epicatechin stimulated cleavage of caspase 3 with or without radiation and this could provide another explanation for radiosensitization in pancreatic cancer cells.

(-)-Epicatechin stimulated mitochondrial respiration and inhibited clonogenic survival in pancreatic cancer cells. When combined with radiation, (-)-epicatechin radiosensitized pancreatic cancer cells and glioblastoma cells, but not HNF cells. (-)-Epicatechin stimulated Chk2 phosphorylation and p21 expression over the level achieved by radiation alone. These findings suggest that (-)-epicatechin is selective in sensitizing cancer cells to radiation and makes it a promising candidate as a novel therapeutic modality for treatment of pancreatic or other cancers.
